# Reforming competitive global public health funding: Bridging equity and practice gaps

**DOI:** 10.1371/journal.pgph.0006588

**Published:** 2026-06-04

**Authors:** Yazoume Ye, Jean Christophe Fotso, Nicolas Meda, Erin Eckert

**Affiliations:** 1 CESMEL Health, Bowie, Maryland, United States of America; 2 Department of International Health and Sustainable Development, School of Public Health and Tropical Medicine, Tulane University, New Orleans, Louisiana, United States of America; 3 EVIHDAF, Opposite Nouvelle Route Omnisport, Yaoundé, Cameroon; 4 Department of Public Health, Faculty of Health Sciences, Université Joseph KI-ZERBO, Ouagadougou, Burkina Faso; 5 Montane RES, LLC, Fort Collins, Colorado, United States of America; PLOS: Public Library of Science, UNITED STATES OF AMERICA

## Introduction

Calls for competitive proposals are among the most influential instruments in global health. They do more than allocate resources: they shape priorities, influence which ideas gain traction, and determine which institutions build capacity. In principle, these mechanisms are designed to promote fairness, transparency, and merit-based selection. In practice, however, they can function as structural gatekeepers, concentrating resources in the Global North while filtering out high-performing but resource-constrained institutions in the Global South.

Proposal development is a routine yet demanding exercise. Applicants are expected to mobilize senior expertise, develop detailed methodologies, assemble partnerships, and produce implementation-ready plans, often under tight timelines and with limited clarity about funding availability or evaluation criteria. The effort is substantial, yet returns are uncertain. Despite this, the model remains largely normalized and rarely questioned.

As major funding bodies, including Gavi and the Global Fund, enter new strategic cycles, there is a need to examine the “wearing friction” embedded in these processes. This need is made more urgent by the current funding environment: recent estimates suggest that development assistance for health fell by 21% between 2024 and 2025 with further declines projected [1], likely to intensify competition for fewer opportunities. At the same time, emerging Artificial Intelligence (AI) tools may also begin to shape proposal competitiveness, with uncertain implications for equity and access [2].

This article explores structural inefficiencies in competitive funding, including proposal labor, opaque commissioning processes, disproportionate requirements, and reliance on proxies of credibility. It also considers legitimate funder constraints and outlines practical reforms to support more efficient and equitable commissioning practices.

## Structural sources of inefficiency

Competitive funding mechanisms are intended to promote innovation and merit-based selection. In practice, their design often generates uneven intellectual and administrative burdens.

First, learning costs are significant. Smaller or newer organizations must invest heavily simply to understand complex and often Western-centric procurement norms. Second, compliance costs are substantial, with due diligence requirements frequently duplicated across funders and for organizations non-English speaking countries, additional burdens may arise from the need to submit and report in English. Third, psychological costs arise when calls appear open but are effectively aligned with incumbent organizations, undermining trust and institutional morale.

Together, these costs contribute substantially to proposal-related research waste. Developing a competitive proposal requires substantial effort, including technical design, budgeting, coordination, and writing. For many organizations, particularly those with limited resources, this process consumes scarce senior staff time and requires time securing local collaborators buy-in. When proposals are unsuccessful, that effort is largely lost, as proposal materials can rarely be reused or repurposed. This represents a significant loss of productive effort, consistent with broader concerns about inefficiencies in the research ecosystem [3–5].

Opacity and uncertainty further amplify these inefficiencies. Limited transparency can undermine predictability, even without bad faith. Some calls are framed as open competitions while appearing aligned with pre-identified partners; others are issued with timelines that favor organizations with standing proposal infrastructure. In some cases, calls are delayed, cancelled, or concluded with minimal feedback after applicants have invested substantial effort. These practices raise concerns in a field that emphasizes equity and stewardship, and contrast with established principles of transparency and proportionality in public procurement [6].

Disproportionate proposal requirements also contribute to systemic inefficiencies. Smaller funding opportunities often require extensive documentation comparable to large-scale programs, while larger calls may begin with shorter concept notes. This imbalance imposes higher relative costs on smaller organizations and may constrain participation based on proposal-writing capacity rather than substantive capability.

## The “Foreign Gaze” and proxies of credibility

A central tension in global health funding lies in the reliance on proxies of credibility, such as institutional reputation and publication history. While these indicators may signal research capacity, they do not necessarily predict implementation effectiveness or contextual relevance.

This dynamic reflects what has been described as the “foreign gaze,” where knowledge systems privilege institutional pedigree over locally grounded expertise [7]. This is not only theoretical; it also influences how proposals are evaluated and funded. In practice, the perceived credibility of a proposal is often linked to the applicant’s ability to navigate complex procurement reporting systems rather than their capacity to deliver results in context. This contributes to persistent disparities in success rates between Northern-led and Southern-led consortia.

More broadly, these dynamics align with concerns about research incentive systems that prioritize measurable outputs over practical value [8]. The result is a system where credibility is often inferred from institutional positioning rather than demonstrated through implementation performance.

These inefficiencies affect not only smaller organizations but also well-resourced institutions. Large universities and international NGOs invest heavily in proposal development, often maintaining dedicated teams to manage the volume and complexity of funding calls. Considerable resources are therefore spent across the system on proposals that may never be funded.

## Recognizing funder constraints and fiduciary realities

Any meaningful discussion of commissioning systems must also acknowledge the realities facing funders. Funding agencies operate under genuine institutional constraints, including unpredictable budgets, shifting priorities, legal requirements, and complex accountability obligations.

Risk aversion, standardized documentation, and compressed timelines often reflect these pressures rather than deliberate exclusion. In challenging operating environments, additional safeguards are necessary to manage fiduciary risk, though they may increase reliance on intermediaries and limit direct engagement with local institutions.

The issue is not that funders act improperly, but that commissioning systems may generate inefficient or inequitable outcomes even in good faith. Reform efforts must recognize these constraints while seeking to improve efficiency and equity

## Toward more responsible commissioning and collective learning

More efficient, equitable commissioning practices are feasible with funder constraints.

First, harmonizing due diligence processes through standards such as Good Financial Grant Practice (GFGP) [9] could significantly reduce administrative burden while strengthening institutional capacity.

Second, fair indirect cost recovery is essential. Many Global South institutions operate within a “nonprofit starvation cycle,” where inadequate overhead funding undermines long-term sustainability [10].

Third, two-stage application processes should become standard practice, beginning with short expressions of interest and inviting full proposals only from shortlisted applicants. This would reduce unnecessary effort while preserving competition.

Fourth, proposal requirements should be proportionate to the funding amount, scope, and complexity of the opportunity.

Fifth, differentiated funding tracks could broaden participation by creating pathways for smaller or emerging institutions.

Sixth, targeted proposal development support for shortlisted applicants may help ensure that strong ideas are not disadvantaged by differences in administrative capacity.

Seventh, greater transparency and collective learning mechanisms are needed. Independent, anonymized platforms documenting applicant experience could generate valuable insights.

Finally, applying political economy analysis to commissioning systems may offer deeper insight into how funding structures shape incentives and institutional behavior.

## Conclusion

Competitive funding remains a central mechanism in global health. The question is not whether competition should exist, but whether it is structured to serve its purpose. Systems that impose high transaction costs, shift uncertainty to applicants, and rely on opaque processes risk undermining efficiency, participation, and trust.

More transparent, proportional, and accountable commissioning practices would reduce wasted effort, broaden engagement, and enhance system credibility. AI may reshape proposal development by reducing drafting and language costs, while introducing inequities where access, oversight, or institutional capacity is uneven. If global health is serious about equity and stewardship, it must extend ethical reflection beyond research and implementation to the design of funding processes themselves.
